# Notes and Notices

**Published:** 1891-03

**Authors:** 


### NOTES AND NOTICES.

ANNOUNCEMENT.—E. B. Treat, Publisher, New York, has in press for early publication the ninth yearly issue of the *International Medical Annual*.

Its corps of thirty-seven editors—specialists in their respective departments, comprising the brightest and best American, English, and French authors—will vie with previous issues in making it even more popular and of more practical value to the medical profession.

We have the assurance of some of the best medical practitioners that the service rendered their profession by this *Annual* cannot be duplicated by any current annual or magazine, and that it is an absolute necessity to every physician who would keep abreast with the continuous progress of practical medical knowledge.

Its *Index of New Remedies* and *Dictionary of New Treatment*, epitomized in one ready reference volume at the low price of $2.75, make it a desirable investment for the busy practitioner, student, and chemist.

IN PRESS.—DIABETES, LECTURES ON—By Robert Saundby, M. D., Edinburgh. 300 8vo pages, $2 75.

IN PRESS.—SEXUAL NEURASTHENIA.—By G. M. Beard, M. D., and A. D. Rockwell, M. D. Third edition, enlarged, $2.75.

THE FIFTH STATE SANITARY CONVENTION of Pennsylvania will be held at Altoona, Friday and Saturday, May 15th, and 16th, 1891, under the auspices of the State Board of Health, assisted by the Board of Health of Altoona and a committee of citizens. This is not in any sense a doctors' convention. All who take an intelligent interest in the promotion of sanitary reform and the protection of the public health are invited not only to be present and take part in the discussions, but to forward to the Secretary, Dr. Benj. Lee, 1532 Pine Street, Philadelphia, for consideration by the Committee of the Board, not later than April 15th, papers on sanitary or hygienic subjects which they would like to present before the convention.

DR. BENJAMIN LEE, Secretary of the State Board of Health of Pennsylvania, has accepted the position of Secretary of the Section on State Medicine of the American Medical Association.

As the meeting takes place in Washington, May 5th, it is important that all papers intended for this Section should be in his hands by the 5th of April. All members of the Association desiring to be enrolled in the Section are requested to forward him their names at 1532 Pine Street, Philadelphia.

THE POST-GRADUATE COURSE.—It is with great pleasure that we chronicle the fact of the establishment of a post-graduate course in connection with the Homœopathic Hospital, of Cleveland, Ohio. It will begin on Tuesday following commencement, and continue two weeks. It will be free to all graduates of the old college, and to others $25. The course will consist of four lectures per day, and the subjects divided among the following:

Surgical Gynæcology, Prof. Biggar; Materia Medica, Prof. Kraft; Physical and Differential Diagnosis, Prof. Pomeroy; Practical Surgery, Prof. J. K. Sanders; Ophthalmology and Otology, Prof. Phillips; Advanced Obstetrics, Prof. J. C. Sanders; Nervous Diseases, Prof. Eggleston; Orificial Surgery, Prof. Wells; Urinary Analysis, Prof. Bishop; Nose and Throat, Prof. Hall.

Upon one day of each week especially obscure and complicated cases will be solicited and examined and treated by the Faculty as a whole.

CORRECTIONS.—February No., page 63, fourth line from top, for laxation read *laxative;* page 63, nineteenth line from top, for then read *thin;* twentieth line from top, after "constipation" insert *with.*

MISSOURI INSTITUTE OF HOMŒOPATHY.—The 15th annual session will be held at Kansas City Tuesday, Wednesday, and Thursday, April 21st, 22d, and 23d, 1891. Officers for 1891: President, T. Griswold Comstock, M. D., St. Louis; 1st Vice-President, H. C. Baker, M. D., Kansas City; 2d Vice-President, W. John Harris, M. D., St. Louis; General Secretary, A. Cuvier Jones, M. D., Holden; Provisional Secretary, L. C. McElwee, M. D., St. Louis; Treasurer, W. B. Morgan, M. D., St. Louis; Board of Censors: W. A. Edmonds, M. D., St. Louis; W. G. Hall, M. D., St. Joseph; A. C. Williamson, M. D., Springfield.

REMOVALS.—Dr. A. O. Pitcher, from Mt. Pleasant, Iowa, to Roanoke, Virginia. Dr. W. S. Hatfield, from Covington, Kentucky, to 278 West Eighth St., Cincinnati, Ohio, where he takes a professor's chair in Pulte Medical College. See his lecture on "Homœopathy" in February No., page 52. Dr. John F. Miller, from 77 West Fiftieth St. to "The Princeton," 324 West Fifty Seventh St., New York. Dr. Wm. C. Richardson, to 3913 North Eleventh St., St. Louis, Mo.

INTERNATIONAL HAHNEMANNIAN ASSOCIATION.—The Secretary, Dr. S. A. Kimball, announces that the coming meeting of the I. H. A. will be held at Richfield Springs, New York, June 23d, 24th, 25th, and 26th. The hotel rates will be $2.50 per day. Information concerning reduced rates on railroads will be given at a later date.

DR. LANDRETH W. THOMPSON, who has been for some years chief of the surgical department of the dispensary of the Hahnemann Medical College and first assistant to the professor of surgery at that institution, has been appointed by the faculty to the post of demonstrator of surgery in that college. The position was made vacant by the resignation of Dr. J. W. Giles, who goes to New York State to take charge of a lucrative practice. Since the opening of the Hahnemann Hospital a few months ago a nurses' training school has been established in that institution, and Dr. Thompson has but recently received the appointment of lecturer upon surgical emergencies and surgical dressing to that department. He is a graduate of the classical department of the University of Pennsylvania and studied surgery under the preceptorship of John E. James, M. D., Professor of Surgery in the Hahnemann College. He has had considerable experience in and devoted a great deal of attention to this branch of his profession and to diseases of the eye and ear. He is of a quiet, retiring manner, but is a hard student, and his advancement is the result of inherent ability. Dr. Thompson has been for years associated with Dr. Bushrod W. James, of Philadelphia, in eye and ear work.

THE CLEVELAND HOMŒOPATHIC HOSPITAL COLLEGE has now a list of fifty-seven registered students for the present session. We wish the college increased success under its new management.

KING'S JOURNAL DIRECTORY FOR 1891, containing a complete list of medical, dental, pharmaceutical, chemical, microscopical, sanitary, veterinary, and medico-legal journals, both home and foreign, is published. Orders should be sent promptly, as the book is sold by subscription only. Price, fifty cents, post-paid. Address, Dr. F. King, Publisher, P. O. Box 587, New York.

The *Directory* will be sent to libraries and managers of advertising departments *free.*

The New Building of Hahnemann Hospital in Philadelphia.—The new building belonging to the Hahnemann Hospital on Fifteenth Street, north of Race, was thrown open to the public Tuesday, October 21st, at ten, and from that time until ten o'clock at night the corridors were filled with a throng of visitors. At twelve M. dedicatory exercises were held in the general clinic operating room with Judge Hanna President of the Board of Trustees. presiding. After a prayer by Dr. McVickar and some music by Mrs. Newkirk, Mrs. Brown, and Mrs. Everest, Rev. Dr. Duhring, son of a homœopathic physician, made the opening address. Dr. Thomas followed with a speech in which he showed the rapid strides the institution has taken since its incorporation in 1871. The exercises were closed by Dr. Newlin standing midway between the new and the old buildings and pronouncing the benediction.

After having been closed for two months, the hospital is now opened for the reception of patients. When closed it had twenty-five beds distributed among the various wards, while now it has between five and six times that many. The building is of pressed brick, with brownstone trimmings, finished entirely in hard wood, practically fireproof, and in every way calculated to be one of the finest and best equipped hospitals in the country. The old building has been refitted for use as a dispensary, in addition to which it has in the basement the electric light plant and most of the steam-heating apparatus. The new building has ten wards—men's medical and surgical, women's medical and surgical, men and women's private, two children's, an isolating, and a gynecological ward. In addition to these, there are about thirty private rooms, four diet kitchens, a dozen bath-rooms, linen closets, nurses' rooms, offices, dining-rooms, board-room, operating-rooms, and medical and surgical lecture-rooms.

Homœopathic Medical Society of the State of Kansas.—Officers for 1890–91: President, M. Jay Brown, Salina; Vice-President, G. H. Anderson, Seneca; Recording Secretary, P. Diederich, Kansas City, Kan.; Corresponding Secretary, D. P. Cook, Clay Center; Treasurer, G. H. T. Johnson, Atchison. Board of Censors: Mrs. F. M. W. Jackson, Emporia; E. R. McIntyre, Topeka; A. M. Hutchinson, Hutchinson. The next meeting will be held at Kansas City, Kansas, commencing the first Wednesday in May, 1891.

International Homœopathic Congress.—The organization and executive management of the Fourth Quinquennial International Homœopathic Congress has been placed in charge of a committee, consisting of the executive committee and eight other members of the American Institute of Homœopathy.

The time appointed for the Congress to meet is June, 1891, and the place selected is Atlantic City, N. J.

In carrying out the duties placed upon them, the committee desire to make such arrangements as will be most acceptable to those who will participate in this Congress, and will best serve the interest of Homœopathy, and contribute to the progress of medical science throughout the world. They hope that every physician will give to it his most active efforts and strongest influence, and that our ablest men will contribute their best thoughts, either in written essays or in personal discussion on the topics selected. The time of this session will be necessarily so limited that many important subjects cannot be properly considered; yet the committee desire to select those which will prove to be of greatest service to the profession, and to have them presented by those most competent to the task, to this end they ask suggestions from those interested.

The usual five days' session of the American Institute of Homœopathy will give place to this Congress. The Institute will assemble, however, on the day preceding the Congress for the transaction of necessary business. The plan now proposed is that the Institute shall hold its session on Tuesday, June 16th, 1891; the Congress will assemble Wednesday, June 17th, and continue one week—namely, Wednesday, Thursday, Friday, Saturday morning (with rest Saturday afternoon and Sunday), Monday and Tuesday; closing on Tuesday, June 23d.

In arranging these many subjects to the best advantage, the committee ask suggestions and assistance from all homœopathic physicians. All communications may be sent to the Chairman, T. Y. Kinne, M. D., Paterson, N. J., or to the Secretary, Pemberton Dudley, M. D., corner Fifteenth and Master Streets Philadelphia.

Fun for Doctors.—Doctor—I have the pleasure of informing you, Mr. Captious, that you are the father of twins.

Mr. C.—Excuse me, doctor, but as there have been so many discrepancies in the census lately I'll have to ask you to oblige me with a recount.—*Boston Courier*.

"A great many people owe their lives to that doctor," said Kicklington.

"Is he an able physician?"

"It isn't exactly that that I referred to. He is never in his office when you want him."—*Washington Post*.

Widower.—"Doctor, your bill is something fearful. After you have doctored my wife to death, you expect me to pay you an enormous bill."

Doctor.—"That's just what I expected you to say. Such a thing as gratitude no longer exists in this world."—*Texas Siftings*.

Miss Gushington.—"Is that Dr. Drake? What a splendid looking man! He's a perfect Achilles."

Uncle George.—"Yes, and like Achilles, he's all right except in his heal."

